# Circular RNA-FK501 binding protein 51 boosts bone marrow mesenchymal stem cell proliferation and osteogenic differentiation via modulating microRNA-205-5p/Runt-associated transcription factor 2 axis

**DOI:** 10.1186/s13018-023-04242-1

**Published:** 2023-10-18

**Authors:** Yingchao Shen, Bo Jiang, Bin Luo, Xiaowei Jiang, Yang Zhang, Qiang Wang

**Affiliations:** 1 Department of Orthopaedics, Changshu Hospital Affiliated to Nanjing University of Chinese Medicine, No. 6 Huanghe Road, Changshu City, 210023 Jiangsu Province China; 2 Department of Hand and Foot Surgery, The Second Affiliated Hospital of Soochow University, Suzhou City, 215004 Jiangsu Province China; 3 School of Biology and Food Engineering, Changshu Institute of Technology, No. 99, South Third Ring Road, Changshu City, 215500 Jiangsu Province China

**Keywords:** Bone marrow mesenchymal stem cell, Circular RNA-FK501 binding protein 51, MicroRNA-205-5p, Runt-related transcription factor2, Proliferation, Osteogenesis differentiation

## Abstract

**Objective:**

Osteogenesis is the key process of bone homeostasis differentiation. Numerous studies have manifested that circular RNA (circRNA) is a critical regulator of osteogenesis. The research was to explore circRNA-mediated mechanisms in osteogenesis.

**Methods:**

Bone marrow mesenchymal stem cells (BMSCs) were cultured and induced to osteogenic differentiation (OD). Then, oe-circ-FKBP5, oe-NC, si-circ-FKBP5, si-NC, miR-205-5p mimic, mimic NC, miR-205-5p inhibitor, inhibitor NC, sh-RUNX2, or sh-NC were transfected into BMSCs. Alkaline phosphatase (ALP) activity was detected by ALP staining, cell mineralization was detected by alizarin red staining, cell proliferation was detected by CCK-8, and cell apoptosis was detected by flow cytometry. Then, the expression of circ-FKBP5, miR-205-5p, RUNX2 and osteogenic marker genes was detected by RT-qPCR, and the expression of RUNX2 protein was detected by Western blot. Finally, the targeting relationship between miR-205-5p and circ-FKBP5 or RUNX2 was verified by bioinformation website analysis and dual luciferase reporter gene detection.

**Results:**

Circ-FK501 binding protein 51 (FKBP5) was distinctly elevated during OD of BMSCs. Elevated circ-FKBP5 boosted the proliferation and OD, as well as expression of osteogenic marker genes while reduced apoptosis of BMSCs. Down-regulation of circ-FKBP5 inhibited BMSCs proliferation, OD and osteogenic marker gene expression, and promoted apoptosis of BMSCs. Subsequently, circ-FKBP5 combined with miR-205-5p and constrained miR-205-5p expression. Silenced miR-205-5p boosted proliferation, OD, and expression of osteogenic marker genes and suppressed apoptosis of BMSCs. However, up-regulation of miR-205-5p inhibited BMSC proliferation, OD and osteogenic marker gene expression, and promoted apoptosis. Additionally, miR-205-5p targeted Runt-associated transcription factor 2 (RUNX2). Repression of RUNX2 turned around the effect of circ-FKBP5 overexpression on BMSCs.

**Conclusion:**

In brief, circ-FKBP5 boosted BMSC proliferation and OD by mediating the miR-205-5p/RUNX2 axis.

**Supplementary information:**

The online version of this article (10.1186/s13018-023-04242-1) contains supplementary material, which is available to authorized users.

## Introduction

Human bone marrow stem cells (BMSCs) are multipotent progenitor cells with self-renewal characteristics and diversified differentiation potential, which are available to differentiate into chondrocytes, adipocytes, and osteoblasts [1]. Differentiation of BMSCs into specific lineages provides opportunities for the therapeutic efficiency of pluripotent cells in regenerative medicine [2]. Furthermore, osteogenic differentiation (OD) of BMSCs has become a prospective treatment strategy for multiple bone diseases [3]. Nevertheless, with age, the ability of BMSCs to differentiate into osteoblasts is reduced, resulting in bone loss [4]. Consequently, realizing the regulatory mechanisms of BMSCs osteoblast differentiation was conducive to developing latent curative strategies.

Circular RNA (circRNA), a new type of non-coding RNA, is generated via reverse splicing of eukaryotic transcriptome and modulates genes via multiple pathways [5]. Unlike linear RNA, circRNA molecules are covalently closed ring structure that is not degraded via RNase [6]. CircRNA is implicated in almost all biological processes, covering OD [7]. Additionally, present studies have proposed a novel post-transcriptional regulatory mechanism, that is, circRNAs/long non-coding RNAs modulate a messenger RNA (mRNA) via competitively combining with microRNA (miRNA), also known as competitive endogenous RNA (ceRNA) theory [8]. Fk506-binding protein 5 (FKBP5) is a member of the immunoaffinity protein family and is involved in a variety of biological processes, including immune regulation, protein folding, and transport [9–11]. As a co-chaperone in the glucocorticoid receptor (GR) complex, FKBP5, together with heat shock protein 90, is involved in the regulation of GR function [12]. It has been proposed that human bone marrow-derived mesenchymal stromal cells are involved in higher gene transcription levels of early osteogenesis/cartilage/adipogenesis (ZNF145, FKBP5) [13]. FKBP5 is the host gene of circ-FKBP5. Nevertheless, the role of circ-FKBP5 in the differentiation of BMSCs osteoblasts remains unclear.

MiRNAs, a group of short endogenous non-coding RNAs with a length of approximately 18–25 nucleotides, modulate post-transcriptional genes via combining with the 3′-untranslated region (UTR) of target mRNA and participate in diversified physiological activities of cells, covering cell differentiation [14]. Presently, miRNAs have been identified as latent regulators of BMSC proliferation, differentiation, and musculoskeletal development [15–19]. For instance, miR-486-3p boosts the OD of BMSCs via targeting catenin beta interacting protein 1 and activating the Wnt/β-catenin pathway [20]. MiR-144-3p constrains BMSC proliferation and OD via targeting frizzled class receptor 4 [21]. MiR-205-5p, a newly authenticated miRNA, is aberrantly expressed during OD [22]. Nevertheless, the latent mechanisms of miR-205-5p in BMSC proliferation and OD have not been completely illustrated.

This study was to explore the action and latent mechanism of circ-FKBP5 in BMSC proliferation and OD. Through in vitro experiments, this research uncovered the molecular mechanism by which circ-FKBP5 accelerated BMSC proliferation and OD via mediating the miR-205-5p/Runt-associated transcription factor 2 (RUNX2) axis. This finding might offer a reference for clinical research on orthopedic diseases.

## Materials and methods

### Cell culture and treatment

Human BMSCs (Shanghai Institutes for Biological Sciences, Shanghai, China) were stored in α-MEM (Sigma-Aldrich, Saint-Louis, Missouri) covering 1% antibiotics and 10% fetal bovine serum (FBS). To stimulate OD, α-MEM osteogenic medium supplemented with 1% antibiotics, 10% FBS, 0.2 mM ascorbic acid, 10 mM β-glycerophosphate, and 100 nM dexamethasone were used.

### Alkaline phosphatase (ALP) staining

ALP staining analysis was implemented in light of the NBT/BCIP kit (CoWin Biotech, Beijing, China). BMSCs were cultured in an osteogenic medium in the 24-well plate for 7, 14, and 21 d, fixed, and incubated with a staining reagent.

### Mineralization test

To detect calcium deposition in the extracellular matrix, BMSCs were incubated with an osteogenic medium in the 24-well plate for 7, 14, and 21 d. After fixation, calcified nodules were treated with 0.1% alizarin red S solution (Sigma-Aldrich) at PH 4.2 [23].

### Cell transfection

To down-regulate circ-FKBP5, small interfering RNA (siRNA) targeting circ-FKBP5 (si-circ-FKBP5) was constructed in U6/GFP/Neo plasmid. oe-circ-FKBP5, oe-NC, si-circ-FKBP5, si-NC, miR-205-5p mimic, mimic NC, miR-205-5p inhibitor, inhibitor NC, sh-RUNX2, and sh-NC were purchased from Genepharma (Shanghai, China). BMSCs were seeded in 2 mL of osteogenic induction medium and transfected using Lipofectamine2000 (Invitrogen). After 24 h of transfection at 37 °C and 5%CO_2_, the previous medium was replaced with a fresh medium and incubated for 48 h [24].

### Cell counting kit-8 (CCK-8)

BMSCs were seeded into 96-well plates (1 × 10^4^ cells/well) and incubated with 10 μL CCK-8 solution (Beyotime) to measure cell proliferation. After 2 h, the absorbance at 450 nm was recorded on a microplate reader.

### Flow cytometry

Apoptotic cells were identified using the fluorescein isothiocyanate (FITC)-Annexin V/propidium iodide (PI) apoptosis kit (BD Biosciences, USA). BMSCs were washed twice with cold PBS and then, resuscitated with 300 μL 1 × binding buffer. Annexin V (10 μL) was added and incubated in the dark at room temperature for 15 min. At the same time, the cells were incubated with 5 μL PI for 5 min and analyzed on a flow cytometer (Thermo, USA) [25].

### Reverse transcription quantitative polymerase chain reaction (RT-qPCR)

Extraction of total RNA from BMSCs was done using TRIzol reagent (Invitrogen; Thermo Fisher Scientific, Inc.), and reverse transcription was performed using PrimeScript RT Reagent Kit (Takara Biotechnology Co., Ltd.). Subsequently, qPCR was performed on ABI 7500 Fast real-time PCR system (Applied Biosystems; Thermo Fisher Scientific, Inc.) with SYBR Green Technology (Takara Biotechnology Co., Ltd.). Glyceraldehyde-3-phosphate dehydrogenase (GAPDH) and U6 were considered loading controls. The 2^−∆∆ct^ method was applied to quantify relative genes. Primer sequences are presented in Table 1.

**Table 1:** RT-qPCR sequences

Genes	Primer sequences (5′– 3′)
circ-FKBP5	F: AGAGCTTCGAAAAGGCCAAAG
	R: CGCCTGCATGTATTTGCCTC
miR-205-5p	F: TCCTTCATTCCACCGGAGTCTG
	R: GCGAGCACAGAATTAATACGAC
RUNX2	F: CCAGATGGGACTGTGGTTACC
	R: ACTT GGTGCAGAGTTCAGGG
ALP	F: GAATCTTCCCCAAGGGCCAA
	R: CAGAATGTTCCACGGAGGCT
OPN	F: GCCGAGGTGATAGTGTGGTT
	R: AACGGGGATGGCCTTGTATG
OCN	F: ACACCATGAGGACCATCTTTC
	R: CGGAGTCTGTTCACTACCTTATT
U6	F: CTCGCTTCGGCAGCACA
	R: AACGCTTCACGAATTTGCGT
GAPDH	F: CACCCACTCCTCCACCTTTG
	R: CCACCACCCTGTTGCTGTAG

### Western blot

Extraction of total protein from BMSCs was carried out using Radio-Immunoprecipitation assay lysis buffer covering protease inhibitors (Sigma-Aldrich; Merck KGaA), and protein concentration was measured using Pierce BCA Protein assay Kit (Thermo Fisher Scientific, Inc.). Subsequently, the total protein extract was separated with 10% sodium dodecyl sulfate–polyacrylamide gel electrophoresis and electroblotted onto a polyvinylidene fluoride membrane. After blocking with 5% skim milk, the membrane was incubated with primary antibodies RUNX2 (AB23981) and GAPDH (AB181602) (both 1: 1000, Abcam) and with horseradish peroxidase-conjugated secondary antibody. Visualization of the bands was implemented using an enhanced chemiluminescence reagent (Invitrogen; Thermo Fisher Scientific, Inc.), and analysis was performed using ImageJ software (National Institutes of Health).

### The luciferase activity assay

After amplification, circ-FKBP5 and RUNX2 sequences were cloned into pmirGLO plasmids (Promega) to gain circ-FKBp5-wild-type (WT) and RUNX2-WT. A fragment covering the mutant target region was designed and cloned into pmirGLO to gain circ-FKBP5-mutant-type (MUT) and RUNX2-MUT. Subsequently, co-transfection of miR-205-5p mimic or mimic NC with circ-FKBP5-MUT or RUNX2-WT or their respective MUT plasmids was done into BMSCs using Lipofectamine 2000. The relative luciferase activity was analyzed after transfection of 48 h in the luciferase reporter gene assay system (Promega) [26].

### RNA pull-down assay

RNA pull-down assay was conducted to validate the interaction between circ-FKBP5 and miR-205-5p using the biotin-LNA-3865 probe (5′-TGGATCTGAATCTGTGTAACT-3′) synthesized by the GENEray company (Shanghai, China) as previously described [27]. BMSCs overexpressing circ-FKBP5 were lysed and incubated with the abovementioned probes for 2 h and "pulled down" with streptavidin-coated magnetic beads (#08014; Sigma-Aldrich). After washing three times with PBS, the eluates were analyzed by quantitative PCR method to detect circ-FKBP5 and miR-205-5p.

### Statistical analysis

Statistical analysis was implemented using GraphPad Prism 8 (Version X). All experiments were conducted in 3 replicates. In this study, student’s t test was employed to analyze differences between groups. Comparison among multiple groups was performed with one-way analysis of variance (ANOVA), and then, the least significant difference test was performed. *P* < 0.05 was accepted as a distinct difference.

## Results

### Circ-FKBP5 is elevated during OD of BMSCs

**Fig. 1:**
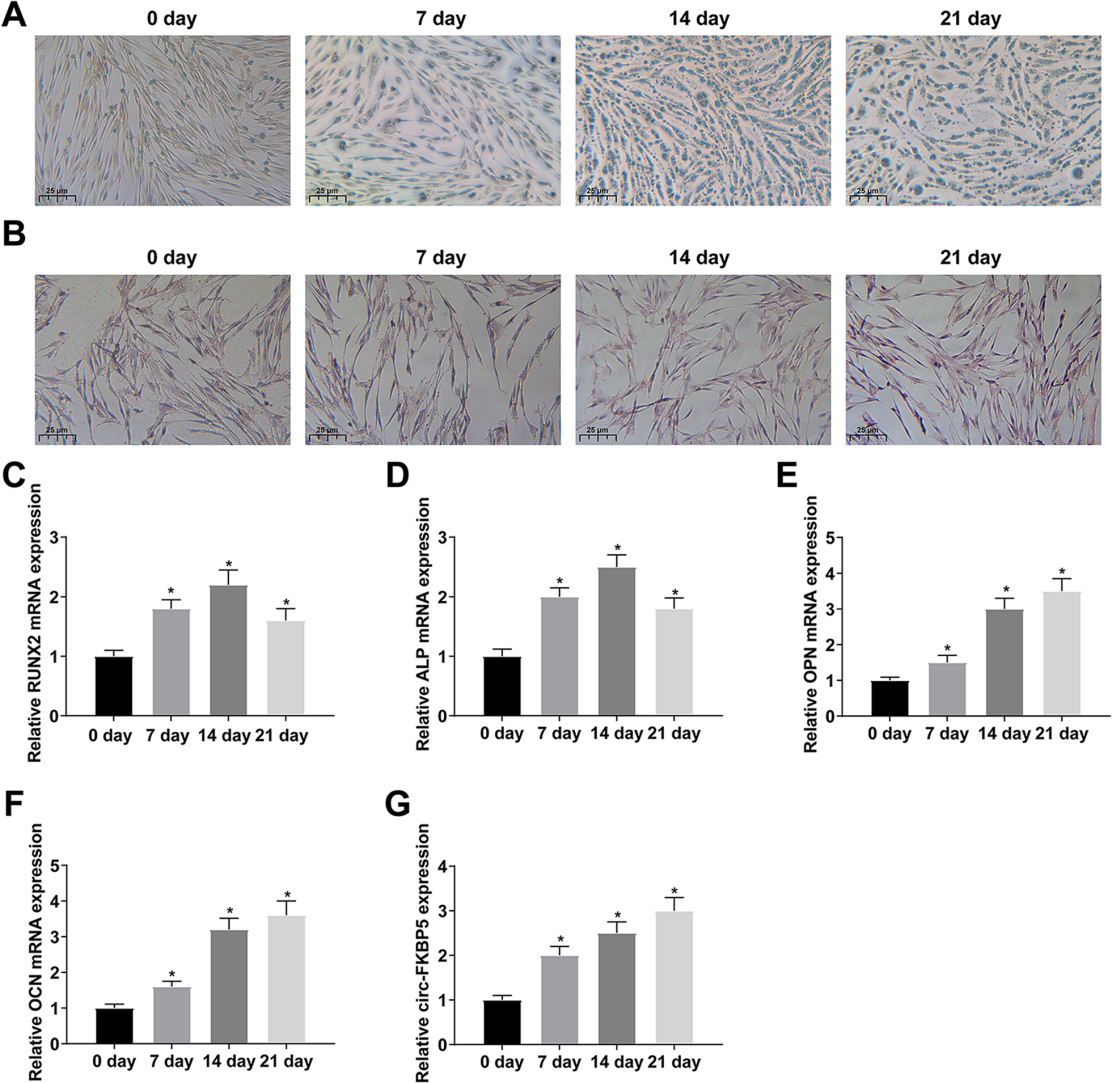
Circ-FKBP5 is elevated during OD of BMSCs. **A**: ALP staining detection of the activity of ALP; **B**: ARS staining test of calcified nodules in BMSCs; **C**–**F**: RT-qPCR test of mRNA of osteogenic markers (RUNX2, ALP, OPN and OCN); **G**: RT-qPCR examination of circ-FKBP5. The data in the figure were all measurement data, and the values were presented as mean ± standard deviation (SD). *Vs. the 0 d, *P* < 0.05

BMSCs were cultured in the osteogenic medium after 7, 14 and 21 d, and OD of BMSCs was detected. The activity of ALP in BMSCs was gradually enhanced after culture (Fig. 1A). The number of calcified nodules in BMSCs was gradually augmented after culture (Fig. 1B). In the meantime, mRNA expressions of RUNX2 and ALP, the early markers of osteogenic differentiation, increased and reached a peak at day 14. The mRNA expression of markers of late osteogenic differentiation (OPN and OCN) increased gradually within 21 days (Fig. 1C–F). To sum up, the OD of BMSCs was successfully stimulated. Additionally, circ-FKBP5 expression was elevated during the OD of BMSCs (Fig. 1G).

### Elevated circ-FKBP5 boosts proliferation and OD of BMSCs

**Fig. 2:**
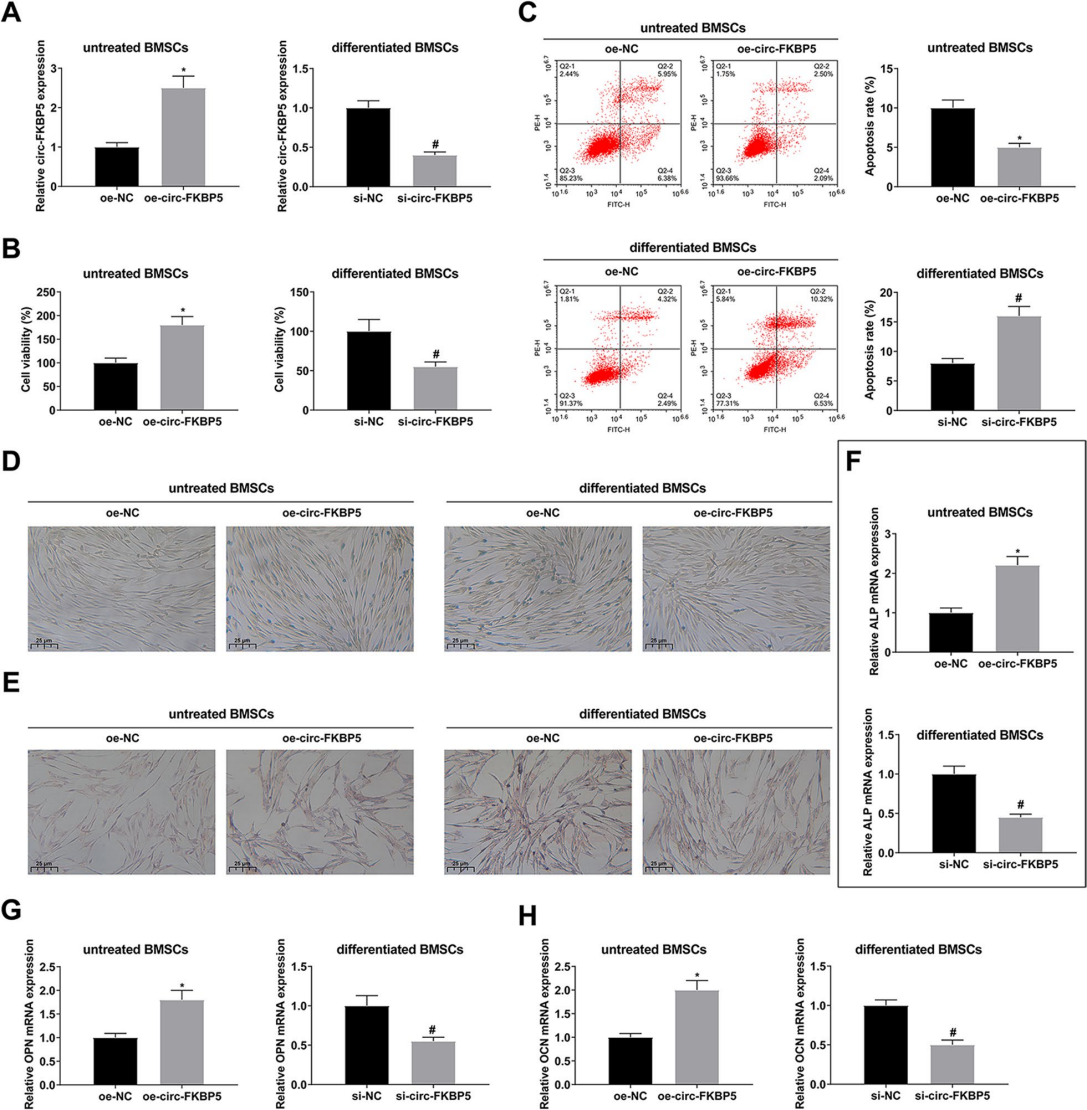
Elevated circ-FKBP5 accelerates proliferation and OD of BMSCs. **A**: RT-qPCR test of circ-FKBP5; **B**: CCK-8 detection of cell viability; **C**: Flow cytometry analysis of apoptosis; **D**: ALP staining test of the activity of ALP; **E**: ARS staining examination of calcified nodules in untreated BMSCs; **F**–**H**: RT-qPCR examination of mRNA of osteogenic markers (ALP, OPN and OCN). The data in the figure were all measurement data, and the values were presented as mean ± SD. *Vs. the oe-NC; *P* < 0.05; ^#^Vs. the si-NC, *P* < 0.05

Whether circ-FKBP5 affects, the OD of BMSCs was explored subsequently. circ-FKBP5 was overexpressed in untreated BMSCs and knocked down in differentiated BMSCs (BMSCs underwent OD for 7 days). The transfection of oe-circ-FKBP5 or si-circ-FKBP5 was verified by RT-qPCR (Fig. 2A). CCK-8 test of cell viability was performed, and the proliferation of untreated BMSCs was elevated by overexpressing circ-FKBP5. In the meantime, silencing circ-FKBP5 declined the proliferation activity of differentiated BMSCs (Fig. 2B). Flow cytometry showed that the apoptosis of untreated BMSCs decreased after up-regulation of circ-FKBP5. However, apoptosis of differentiated BMSCs increased after circ-FKBP5 was down-regulated (Fig. 2C). Elevated circ-FKBP5 augmented the activity of ALP and accelerated the formation of calcified nodules in untreated BMSCs, while repression of circ-FKBP5 exerted an opposite influence on differentiated BMSCs (Fig. 2D–E). Additionally, elevated circ-FKBP5 augmented the mRNA expression of osteogenic markers in BMSCs, while suppression of circ-FKBP5 declined the mRNA expression of osteogenic genes in differentiated BMSCs (Fig. 2F–H). In short, elevated circ-FKBP5 boosted the proliferation and OD of BMSCs.

### Circ-FKBP5 positively modulates RUNX2 as ceRNA of miR-205-5p

**Fig. 3:**
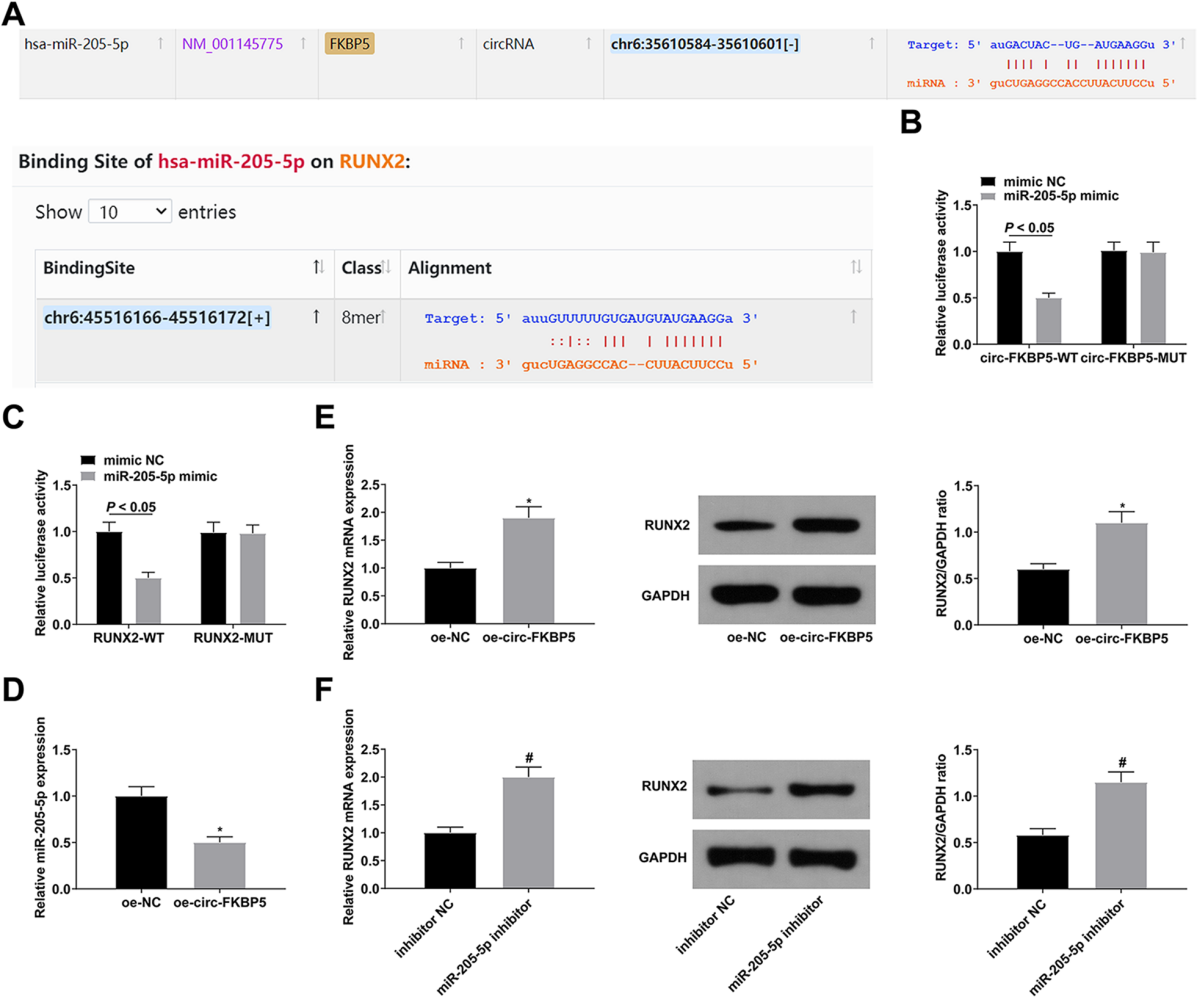
Circ-FKBP5 positively mediates RUNX2 as a ceRNA of miR-205-5p. **A**: Bioinformatics analysis’ prediction of binding sites of miR-205-5p with circ-FKBP5 and RUNX2; **B**–**C**: The luciferase activity verification of the binding of miR-205-5p with circ-FKBP5 and RUNX2; **D**–**E**: RT-qPCR or Western blot test of miR-205-5p and RUNX2 after elevating circ-FKBP5; **F**: RT-qPCR or Western blot examination of RUNX2 after transfection with miR-205-5p inhibitor. The data in the figure were all measurement data, and the values were presented as mean ± SD. *Vs. the si-NC, *P* < 0.05; ^#^Vs. the inhibitor NC, *P* < 0.05

To further research the mechanism of circ-FKBP5 in OD, miRNA targets of circ-FKBP5 were predicted via the bioinformation website starBase (http://www.sysu.edu.cn/403.html). circ-FKBP5 had a binding site of miR-205-5p, and miR-205-5p targeted the 3’UTR of RUNX2 (the critical regulator of OD) (Fig. 3A). In addition, several studies have shown that miR-205-5p targets RUNX2 [22, 28]. Therefore, miR-205-5p and its target RUNX2 were selected for further study. The relative luciferase activity was suppressed after co-transfection with miR-205-5p mimic and circ-FKBP5-WT, while co-transfection with miR-205-5p mimic and RUNX2-WT also reduced the luciferase activity (Fig. 3B–C). RNA pull-down assay further demonstrated the interaction between circ-FKBP5 and miR-205-5p (Additional file 1: Fig. S1). Additionally, RT-qPCR results found that overexpression of circ-FKBP5 significantly reduced the expression of miR-205-5p and increased the expression of RUNX2 mRNA and protein (Fig. 3D–E). Likewise, RUNX2 expression was elevated after repressing miR-205-5p (Fig. 3F). All in all, circ-FKBP5 is directly combined with miR-205-5p to mediate RUNX2 in BMSCs.

### Suppressing miR-205-5p boosts proliferation and OD of BMSCs

**Fig. 4:**
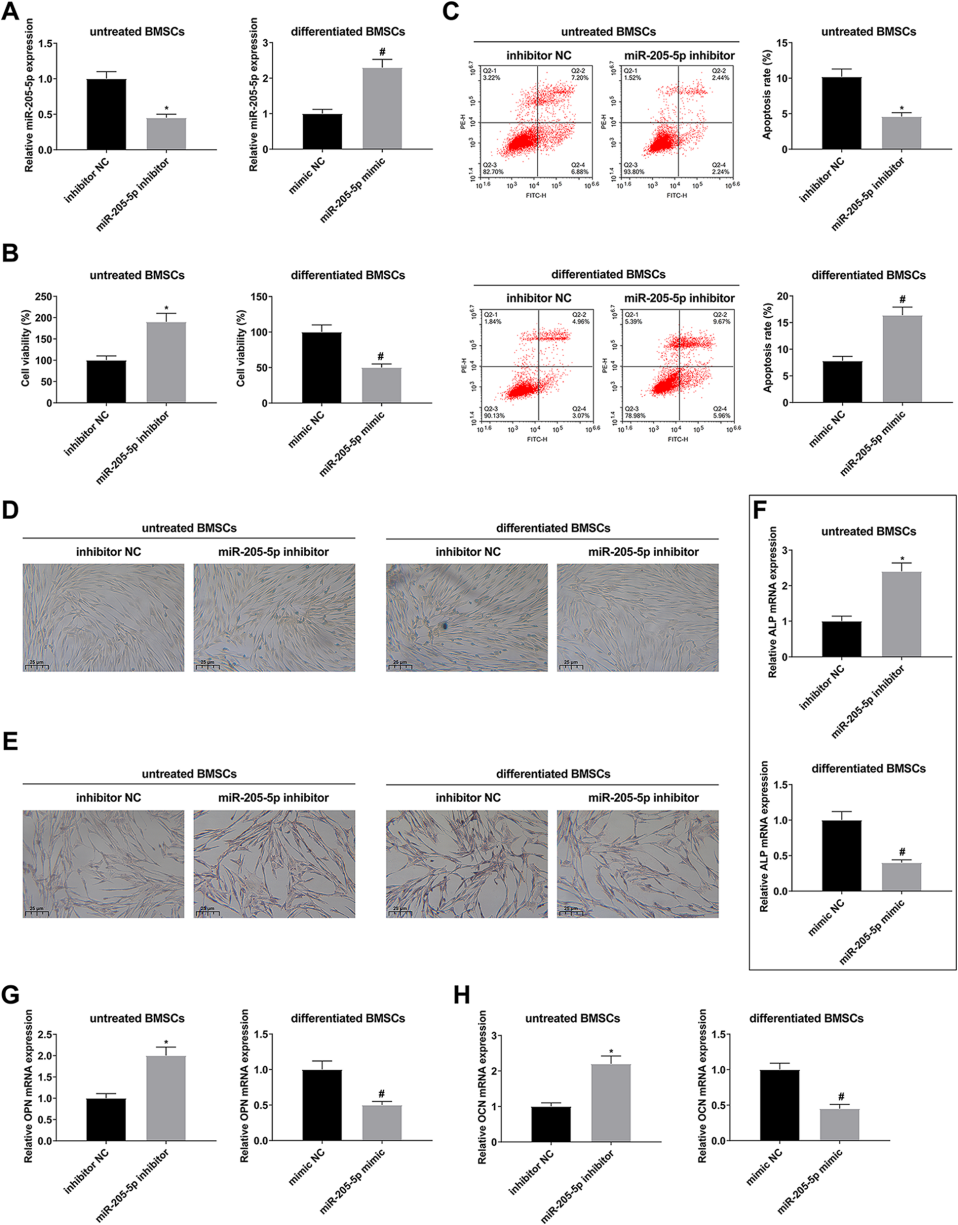
Silenced miR-205-5p boosts proliferation and OD of BMSCs. **A**: RT-qPCR detection of miR-205-5p; **B**: CCK-8 test of cell viability; **C**: Flow cytometry analysis of apoptosis; **D**: ALP staining examination of the activity of ALP; **E**: ARS staining detection of calcified nodules in untreated BMSCs; **F**–**H**: RT-qPCR examination of mRNA of osteogenic markers (ALP, OPN and OCN). The data in the figure were all measurement data, and the values were presented as mean ± SD. *Vs. the inhibitor NC; *P* < 0.05; ^#^Vs. the mimic NC, *P* < 0.05

The impacts of miR-205-5p on the OD of BMSCs were explored. MiR-205-5p was silenced in untreated BMSCs, and miR-205-5p was elevated in differentiated BMSCs (BMSCs underwent OD for 7 d) (Fig. 4A). The proliferation activity of untreated BMSCs was augmented after silencing miR-205-5p, while that of differentiated BMSCs was repressed after augmenting miR-205-5p (Fig. 4B). Flow cytometry showed that apoptosis of untreated BMSCs was decreased after down-regulation of miR-205-5p. However, apoptosis of differentiated BMSCs increased after up-regulation of miR-205-5p (Fig. 4C). Silenced miR-205-5p elevated the activity of ALP and boosted the formation of calcified nodules in untreated BMSCs, while overexpressed miR-205-5p exerted the opposite impacts on differentiated BMSCs (Fig. 4D–E). Additionally, silenced miR-205-5p elevated mRNA expression of osteogenic markers in untreated BMSCs, while augmented miR-205-5p declined mRNA expression of osteogenic genes in differentiated BMSCs (Fig. 4F–H). In general, silenced miR-205-5p boosted the proliferation and OD of BMSCs.

### RUNX2 down-regulation turns around the effect of up-regulation of circ-FKBP5 on BMSCs

**Fig. 5:**
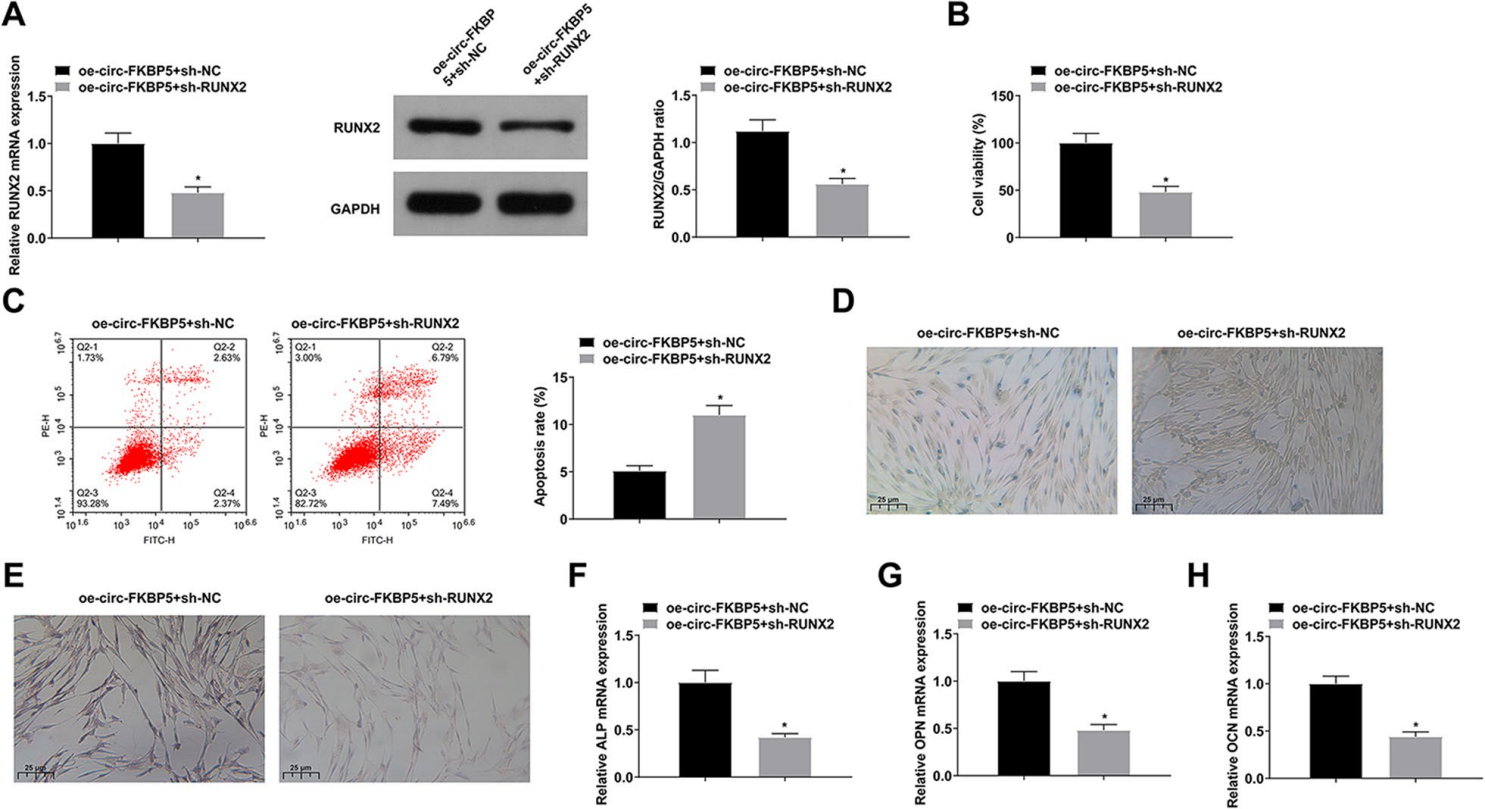
Silenced RUNX2 turns around the impacts of up-regulation of circ-FKBP5 on BMSCs. **A**: RT-qPCR and Western blot detection of RUNX2; **B**: CCK-8 test of cell viability; **C**: Flow cytometry analysis of apoptosis; **D**: ALP staining detection of the activity of ALP; **E**: ARS staining examination of calcified nodules; **F**–**H**: RT-qPCR test of mRNA of osteogenic markers (ALP, OPN and OCN). The data in the figure were all measurement data, and the values were presented as mean ± SD. *Vs the oe-circ-FKBP5 + sh-NC; *P* < 0.05

To functionally verify the role of circ-FKBP5/miR-205-5p/RUNX2 regulatory axis in BMSCs, transfection of oe-circ-FKBP5 + sh-RUNX2 or oe-circ-FKBP5 + sh-NC was performed in untreated BMSCs. sh-RUNX2 turned around the effect of oe-circ-FKBP5 on RUNX2 expression (Fig. 5A). oe-circ-FKBP5-triggered BMSCs proliferation, BMSCs apoptosis, ALP activity, and formation of calcified nodules were eliminated after silencing RUNX2 (Fig. 5B–E). In the meantime, silenced RUNX2 turned around the effect of elevated circ-FKBP on mRNA expression of osteogenic markers (Fig. 5F–H). In short, circ-FKBP5 boosted BMSC proliferation and OD by modulating the miR-205-5p/RUNX2 axis.

## Discussion

Dysfunction of OD of BMSCs is the critical cause of bone loss and osteoporosis [20]. Numerous studies have illuminated that non-coding RNA is a regulatory factor in the OD of BMSCs [29, 30]. CircRNA, a special non-coding RNA, broadly exists in epigenetic, transcription and post-transcriptional genes, exerting a critical action in various biological activities and performing as a latent biomarker for diversified types of diseases [31, 32]. Moreover, the recent evidence has studied the role of ncRNAs in musculoskeletal conditions [16–19]. In this research, circ-FKBP5 was up-regulated during OD of BMSCs, and elevated circ-FKBP5 boosted proliferation and OD of BMSCs in vitro, while knockdown of circ-FKBP5 did oppositely. These results clarified that circ-FKBP5 was a positive regulator of BMSC OD.

The OD of BMSCs is a complex process covering multiple signaling pathways like NF-κB [33], Wnt [34] and PI3K/Akt pathway [35]. Numerous studies have elucidated that circRNA exerts a crucial role in the OD of BMSCs. For instance, circ-DAB1 accelerated cell proliferation and OD of BMSC via the RBPJ/DAB1 axis [36]. Circ_0066523 boosts the proliferation and differentiation of BMSCs via repressing PTEN to activate the AKT pathway [37]. Additionally, circRNA is also available to modulate the OD of BMSCs via performing as a sponge of miRNA to competitively combine with miRNA. For instance, circ_0062582 boosts the OD of BMSCs via mediating miR-145/core-binding factor subunit β axis [38]. Hsa_circ_0006766 accelerates OD of BMSCs via targeting miR-4739/Notch2 axis [39]. In this research, circ-FKBP5 was elevated during BMSC OD, suggesting that circ-FKBP5 might be implicated in the modulation of BMSC OD. Additionally, in vitro cell test results manifested that overexpressing circ-FKBP5 boosted proliferation, OD, and osteogenic marker gene expression, and suppressed apoptosis of BMSCs. On the contrary, silenced circ-FKBP5 worked oppositely.

CircRNAs are ceRNA with miRNA response elements that function as miRNA sponges. The absence or presence of ceRNA regulates gene expression by affecting miRNA functional activity [40]. As a key regulator of gene expression at a post-transcriptional level, miRNAs can bind to complementary gene sites according to the principle of base complementarity, and the changes in miRNAs can regulate the expression of genes and proteins. Complementary circRNAs can bind to their target miRNAs and inhibit their function. We confirmed for the first time that circ-FKBP5 directly binds miR-205-5p and inhibits its expression by starBase prediction, dual luciferase reporter assay, and RNA pull-down assay. A foregoing study elaborated that miR-205-5p expression was suppressed when OD and osteoblast differentiation were both constrained via targeting RUNX2 [22]. In this research, miR-205-5p was down-regulated in OD of BMSCs, and elevated miR-205-5p repressed proliferation, OD, and osteogenic marker gene expression of BMSCs in vitro, and promoted apoptosis of BMSCs, while silenced miR-205-5p led to the opposite results. Additionally, miR-205-5p targeted RUNX2 and mediated RUNX2 expression.

RUNX2, an indispensable transcription factor for bone development, encodes a nuclear protein and controls osteoblast differentiation and bone formation [41]. RUNX2 modulates its activity, stability and interactions with transcriptional coregulators and chromatin remodeling proteins of osteogenic signal downstream via post-translation of phosphorylation, ubiquitination and acetylation [42]. RUNX2 is augmented during OD [43], and calcium accumulation and activity of ALP in cells were distinctly stimulated [44]. Suppression of RUNX2 constrained osteogenesis and osteoblast differentiation [45]. Additionally, RUNX2 is modulated via miRNA. For instance, miR-217 constrains the OD of BMSCs via binding to Runx2 [46]. It is testified that RUNX2 is elevated during OD of BMSCs, and silenced RUNX2 turned around the effect of circ-FKBP5 overexpression on OD of BMSCs. These results clarified that circ-FKBP5 accelerated proliferation, OD, and osteogenic marker gene expression of BMSCs and inhibited apoptosis of BMSCs via modulating the miR-205-5p/RUNX2 axis.

Nevertheless, several limitations are presented in the study. First of all, this study only explored the influence of circ-FKBP5 on the OD of BMSCs, and the impact of circ-FKBP5 on OD of mesenchymal cells from other sources like adipose-derived mesenchymal cells was not analyzed. Additionally, in vivo experiments should be further carried out to support the viewpoint that the circ-FKBP5/miR-205-5p/RUNX2 axis accelerated the proliferation and OD of BMSCs. In later research, in vivo experiments should be conducted to verify the function of the circ-FKBP5/miR-205-5p/RUNX2 axis in osteogenesis, which is also the emphasis for later work.

## Conclusion

In brief, circ-FKBP5 is elevated during the OD of BMSCs and boosts RUNX2 via absorbing miR-205-5p, thereby accelerating the proliferation, OD, and osteogenic marker gene expression of BMSCs and reduced apoptosis of BMSCs in vitro. These results provide new insights into the molecular mechanisms of osteogenesis, thereby revealing novel strategies to promote OD of BMSCs by promoting circ-FKBP5 or inhibiting miR-205-5p.

### Supplementary information

**Additional file 1.Fig. S1**: Interaction between circ-FKBP5 and miR-205-5p. RNA pull-down assay further demonstrated the interaction between circ-FKBP5 and miR-205-5p. Data in the figure are measurement data, and values are expressed as mean ± SD.

## Data Availability

Data are available from the corresponding author on request.
