# Supplementary material for: Circular RNA-FK501 binding protein 51 boosts bone marrow mesenchymal stem cell proliferation and osteogenic differentiation via modulating microRNA-205-5p/Runt-associated transcription factor 2 axis
Source: J Orthop Surg Res. 2023 Oct 18;18:782. doi: 10.1186/s13018-023-04242-1 (PMC10583363; doi:10.1186/s13018-023-04242-1)
Supplement: Supplementary file 1 — Additional file 1.Fig. S1: Interaction between circ-FKBP5 and miR-205-5p. RNA pull-down assay further demonstrated the interaction between circ-FKBP5 and miR-205-5p. Data in the figure are measurement data, and values are expressed as mean ± SD. [file 13018_2023_4242_MOESM1_ESM.docx]

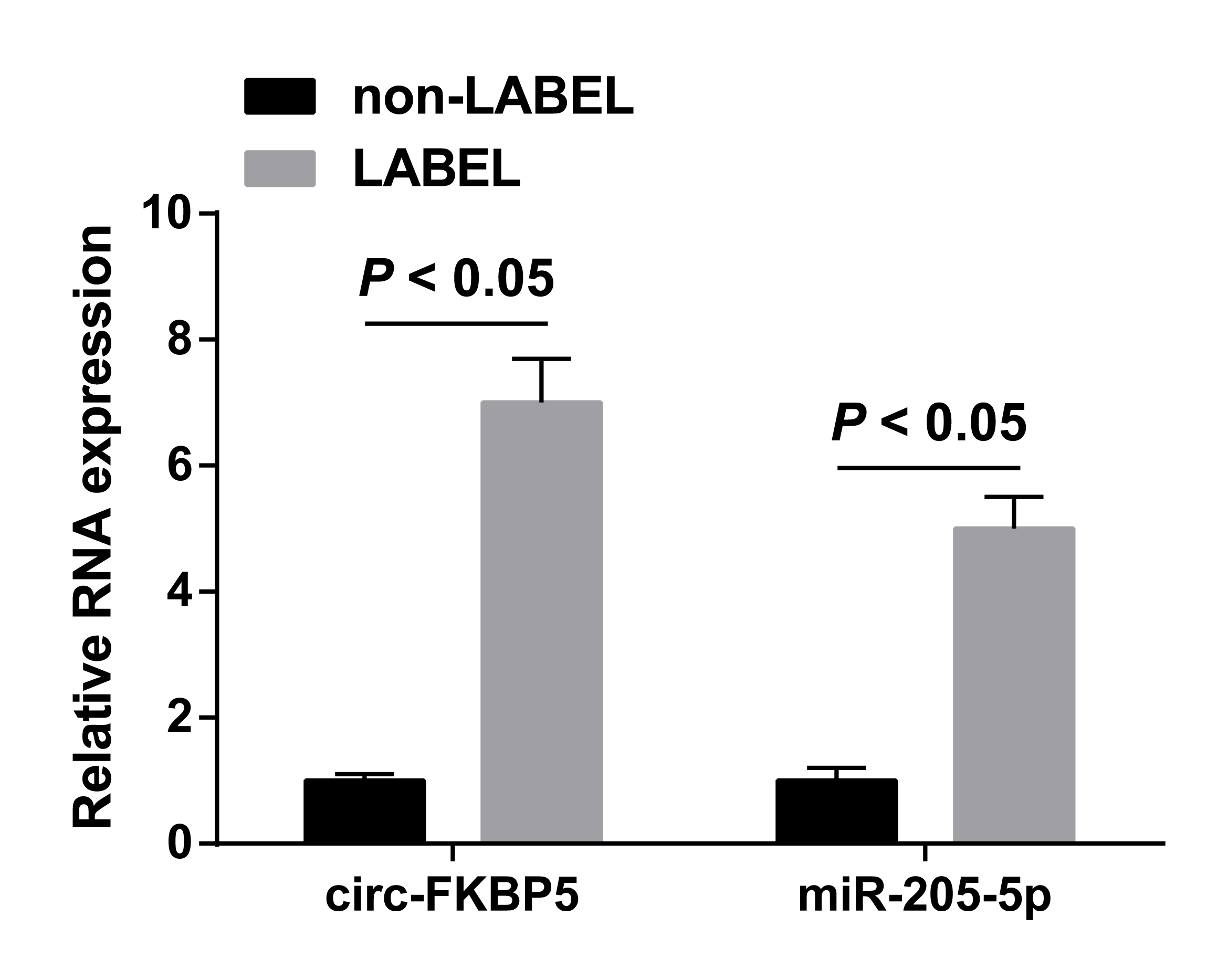


**Supplementary Fig. 1 Interaction between circ-FKBP5 and miR-205-5p**

RNA pull-down assay further demonstrated the interaction between circ-FKBP5 and miR-205-5p. Data in the figure are measurement data, and values are expressed as mean ± SD.
